# The relationship between frailty, nutritional status, co-morbidity, CT-body composition and systemic inflammation in patients with COVID-19

**DOI:** 10.1186/s12967-022-03300-2

**Published:** 2022-02-21

**Authors:** Josh McGovern, Yassir Al-Azzawi, Olivia Kemp, Peter Moffitt, Conor Richards, Ross D. Dolan, Barry J. Laird, Donald C. McMillan, Donogh Maguire

**Affiliations:** 1 Academic Unit of Surgery, University of Glasgow, Level 2, New Lister Building, Glasgow, G31 2ER UK; 2 Emergency Department, Glasgow Royal Infirmary, Glasgow, G4 0SF UK; 3 Institute of Genetics and Molecular Medicine, University of Edinburgh, Edinburgh, EH4 2XU UK

**Keywords:** Frailty, Elderly, COVID-19, Body composition

## Abstract

**Background:**

Frailty, determined by the Canadian Study of Health and Aging-Clinical Frailty Scale (CFS), is strongly associated with clinical outcomes including mortality in patients with COVID-19. However, the relationship between frailty and other recognised prognostic factors including age, nutritional status, obesity, sarcopenia and systemic inflammation is poorly understood. Therefore, the aim of this study was to examine the relationship between frailty and other prognostic domains, in patients admitted with COVID-19.

**Methods:**

Patients who presented to our institutions between 1st April 2020–6th July 2020 with confirmed COVID-19 were assessed for inclusion. Data collected included general demographic details, clinicopathological variables, CFS admission assessment, Malnutrition Universal Screening Tool (MUST), CT-BC measurements and markers of systemic inflammation.

**Results:**

106 patients met the study inclusion criteria. The majority of patients were aged ≥ 70 years (67%), male (53%) and frail (scoring > 3 on the CFS, 72%). The majority of patients were not malnourished (MUST 0, 58%), had ≥ 1 co-morbidity (87%), were sarcopenic (low SMI, 80%) and had systemic inflammation (mGPS ≥ 1, 81%, NLR > 5, 55%). On multivariate binary logistics regression analysis, age (p < 0.01), COPD (p < 0.05) and NLR (p < 0.05) remained independently associated with frailty. On univariate binary logistics regression, NLR (p < 0.05) was significantly associated with 30-day mortality.

**Conclusion:**

Frailty was independently associated with age, co-morbidity, and systemic inflammation. The basis of the relationship between frailty and clinical outcomes in COVID-19 requires further study.

*Trial registration* Registered with clinicaltrials.gov (NCT04484545)

## Introduction

The World Health Organization (WHO) declared the outbreak of novel coronavirus 19 (COVID-19) a global pandemic on the 11th of March 2020 [1]. During the first wave of the pandemic, the Canadian Study of Health and Aging-Clinical Frailty Scale (CFS) [2] was utilised to aid decisions on treatment escalation and ceiling of care for patients admitted with COVID-19 [3].

The CFS is a clinical judgement-based frailty tool that evaluates specific domains including comorbidity, function, and cognition [2]. Recent systematic reviews have shown that frailty, determined by the CFS, is associated with clinical outcomes in patients with COVID-19, regardless of age [4]. Furthermore, large multi-centre cohort studies have shown that a high CFS was independently associated with 30-day mortality [5]. These observations are consistent with recent cohort studies from our own population, that have shown a CFS > 3 was independently associated with 30-day mortality in patients admitted to hospital with COVID-19 [6, 7].

However, a recent cohort study (n = 1071) comparing the effect of frailty on 30-day mortality in COVID-19 positive and COVID-19 negative older patients (> 65 years), reported that frailty made only a small contribution to the hazard of dying in patients admitted with COVID-19 [8]. Furthermore, independent of age, comorbidity including obesity, sarcopenia and systemic inflammation are also associated with worsened clinical outcomes [7, 9]. At present, the basis of the relationship between age, frailty and clinical outcomes is not clear. Specifically, the relationship between frailty and other recognised prognostic factors in COVID-19 is unknown. Therefore, the aim of the present study was to examine the relationship between frailty, nutritional status, CT-body composition and systemic inflammation, in patients with COVID-19.

## Methods

A retrospective analysis of prospectively collected data on patients who presented to Glasgow Royal Infirmary or the Queen Elizabeth University Hospital, Glasgow, UK, between the 1st April 2020–6th July 2020 was carried out. In line with NHS policy, this study was approved by the NHS Greater Glasgow and Clyde Caldicott guardian. The study protocol (GN20AE307) was approved by the North West England—Preston research ethics committee (20/NW/0336) and registered with clinicaltrials.gov (NCT04484545).

Patients with either a positive polymerase chain reaction (PCR) test or radiological changes characteristic of COVID-19 infection, reported on chest X-ray (CXR) or CT thorax, by a board-certified radiologist were assessed for inclusion in the study. Exclusion criteria were as follows; patients with cross sectional scanning at the level other the third lumbar vertebra, had CT imaging out with 3 months of the diagnosis with COVID-19 or had CT imaging with significant movement artefact or missing region of interest.

Routine demographic details, clinico-pathological characteristics, frailty and nutritional assessments, as well as haematological and biochemical laboratory results were recorded. Age, sex, BMI and diagnostic modality confirming COVID-19 infection, as well as date of diagnosis, were minimal inclusion criteria. Age categories were grouped to </≥ 70 years. BMI was categorised as ≤ 25/> 25 kg/m^2^. Co-morbidity data collected included a diagnosis of hypertension, heart failure, chronic obstructive pulmonary disease, type 2 diabetes mellitus, liver disease, chronic kidney disease and active cancer. Frailty was assessed using the 9-catergory Clinical Frailty Scale (CFS) [2]. Malnutrition was screened using the five-step Malnutrition Universal Screening Tool (MUST) [10]. Both frailty and MUST scores were identified from admission nursing assessments. Patients with CFS > 3 were categorized as frail. Patients were classified as no risk (MUST = 0), or at risk of malnutrition (MUST ≥ 1). Admission serum C-reactive protein (CRP), albumin and differential blood cell counts were categorised using local reference intervals. An autoanalyzer was used to measure serum CRP (mg/L) and albumin (g/L) concentrations (Architect; Abbot Diagnostics, Maidenhead, UK). Systemic inflammation was determined using Neutrophil/lymphocyte ratio (NLR) [11] and the modified Glasgow Prognostic Score (mGPS) [12]. For this study, thresholds of NLR < 3, 3–5 and > 5 were chosen. mGPS values were grouped as 0, 1 and 2.

### Body composition analysis

Each CT image was individually analysed with ImageJ—a free to download, Java-based program developed by NIH (NIH ImageJ version 1.47, http://rsbweb.nih.gov/ij/) shown to provide reliable measurements [13]—using our departmental standardized methodology [14, 15]. Body composition measurements derived from the CT image slice at L3 included total fat area (TFA), visceral fat area (VFA), and skeletal muscle area (SMA). Attenuation thresholds were from − 190 to + 30 Hounsfield units (HU) for fat and − 29 to + 150 HU for muscle. Skeletal muscle radiodensity (SMD, HU) was calculated as the mean of the measured muscle area used to calculate SMI. Subcutaneous fat area (SFA) was calculated by subtraction of the VFA from TFA. SFA and SMA measurements were then normalized by division of the patient’s height in meter squared to generate subcutaneous fat index (SFI, cm^2^/m^2^) and skeletal muscle index (SMI, cm^2^/m^2^). These indices were then compared with established thresholds for body composition status [16–18].

### Statistical analysis

Demographic data, clinico-pathological variables, CFS, MUST score, CT body composition measurements, mGPS and NLR were presented as categorical variables. Categorical variables were analysed using χ^2^ test for linear-by-linear association.

Associations between CFS and demographic data, clinicopathological variables, MUST score, CT body composition measurements, mGPS and NLR were analysed using univariate and a multivariate backward conditional approach. A *p* < 0.05 was applied to inclusion at each step in the multivariate analysis.

Missing data were excluded from analysis on a variable-by-variable basis. Two-tailed *p* values < 0.05 were considered statistically significant. Statistical analysis was performed using SPSS software version 25.0. (SPSS Inc., Chicago, IL, USA).

## Results

**Fig. 1:**
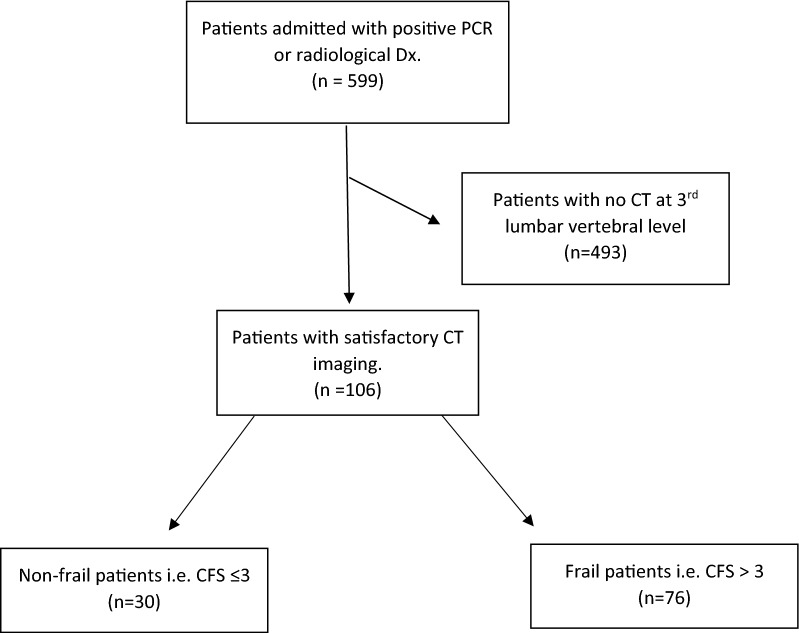
Flow diagram of included patients with COVID 19 Infection and analysable CT imaging

Of the 599 patients admitted during the study period, 106 met the study inclusion criteria (See Fig. 1). The clinicopathological characteristics at presentation are shown in Table 1. The majority of patients were aged ≥ 70 years (67%), male (53%), frail (scoring > 3 on the CFS, 72%) and overweight (BMI > 25, 55%). Furthermore, the majority of patients were not malnourished (MUST 0, 73%), had ≥ 1 co-morbidity (87%), were sarcopenic (low SMI, 80%) and had systemic inflammation (mGPS > 0, 81%, NLR > 5, 55%).

**Table 1:** The relationship between clinicopathological characteristics, MUST, CT-BC and systemic inflammation in patients with COVID-19 as stratified by CFS

Clinicopathological	All (n = 106)	Non Frail (CFS ≤ 3) n = 30 (28.3%)	Frail (CFS > 3) n = 76 (71.7%)	P value^a^
Sex				0.714
Male	56 (52.8)	15 (50)	41 (53.9)	
Female	50 (47.2)	15 (50)	35 (46.1)	
Age (years)				< 0.001
< 70	35 (33.0)	18 (60.0)	17 (22.4)	
≥ 70	71 (67.0)	12 (40.0)	59 (77.6)	
Smoking Hx.				0.145
Yes	68 (64.2)	16 (53.3)	52 (68.4)	
No	38 (35.8)	14 (47.7)	24 (31.6)	
Alcohol excess Hx.				0.229
Yes	18 (17.0)	3 (10.0)	15 (19.7)	
No	88 (83.0)	27 (90.0)	61 (80.3)	
MUST^b^				0.380
0	53 (57.6)	12 (50.0)	41 (60.3)	
≥ 1	39 (42.4)	12 (50.0)	27 (39.7)	
Hypertension				0.006
Yes	47 (44.3)	7 (23.3)	40 (52.6)	
No	59 (55.7)	23 (76.7)	36 (47.4)	
Heart failure				0.211
Yes	14 (13.2)	2 (6.7)	12 (15.8)	
No	92 (86.8)	28 (93.3)	64 (84.2)	
COPD				0.005
Yes	22 (20.8)	1 (3.3)	21 (27.6)	
No	84 (79.2)	29 (96.7)	55 (72.4)	
T2DM				0.043
Yes	33 (31.1)	5 (16.7)	28 (36.4)	
No	73 (68.9)	25 (83.3)	48 (63.2)	
Liver disease				0.270
Yes	13 (12.3)	2 (6.7)	11 (14.5)	
No	93 (87.7)	28 (93.3)	65 (85.5)	
Chronic renal failure				0.514
Yes	22 (20.8)	5 (4.7)	17 (22.4)	
No	84 (79.2)	25 (83.3)	59 (77.6)	
Active cancer				0.545
Yes	17 (16.0)	4 (13.3)	13 (17.1)	
No	89 (84.0)	26 (86.7)	63 (82.9)	
Anaemia				0.065
Yes	54 (50.9)	11 (36.7)	43 (56.6)	
No	52 (49.1)	19 (63.3)	33 (43.4)	
BMI (kg/m^2^)				0.492
≤ 25	48 (45.3)	12 (40.0)	36 (47.4)	
> 25	58 (54.7)	18 (60.0)	40 (52.6)	
High SFI (cm^2^/m^2^)				0.072
Yes	79 (74.5)	26 (86.7)	53 (69.7)	
No	27 (25.5)	4 (13.3)	23 (30.3)	
High VFA (cm^2^)				0.965
Yes	71 (67.0)	20 (66.7)	51 (67.1)	
No	35 (33.0)	10 (33.3)	25 (32.9)	
Low SMI (cm^2^/m^2^)				0.610
Yes	85 (80.2)	25 (83.3)	60 (78.9)	
No	21 (19.8)	5 (16.7)	16 (21.1)	
Low SMD (HU)				0.346
Yes	84 (79.2)	22 (73.3)	62 (81.6)	
No	22 (20.8)	8 (26.7)	14 (18.4)	
CRP (mg/L)				0.976
≤ 150	85 (80.2)	24 (80.0)	61 (80.3)	
> 150	21 (19.8)	6 (20.0)	15 (19.7)	
Albumin (g/L)				0.716
≥ 25	86 (81.1)	25 (83.3)	61 (80.3)	
< 25	20 (18.9)	5 (16.7)	15 (19.7)	
NLR				0.003
< 3	25 (23.6)	11 (36.7)	14 (18.4)	
3–5	23 (21.7)	10 (33.3)	13 (17.1)	
> 5	58 (54.7)	9 (8.5)	49 (64.5)	
mGPS				0.278
0	20 (18.9)	6 (20.0)	14 (18.4)	
1	13 (12.3)	7 (23.3)	6 (7.9)	
2	73 (68.9)	17 (56.7)	56 (73.7)	

^a^P value from χ^2^ analysis

^b^14 patients had no documented nutritional asessment on admission

### Relationship between CFS, clinico-pathological characteristics, MUST, CT-BC and systemic inflammation

On univariate analysis, a CFS > 3, was associated with age (p < 0.001), hypertension (p < 0.01), COPD (p < 0.01), type 2 diabetes mellitus (p < 0.05), anaemia (p < 0.10), high SFI (p < 0.10) and NLR (p < 0.01). Frailty was not associated with sex (p = 0.714), smoking history (p = 0.145), excessive alcohol consumption history (p = 0.229), MUST (p = 0.380), heart failure (p = 0.211), liver disease (p = 0.270), chronic renal failure (p = 0.514), active cancer (p = 0.545), BMI > 25 (p = 0.492), high VFA (p = 0.965), low SMI (p = 0.610), low SMD (p = 0.346) or mGPS (p = 0. 278, see Table 1).

On multivariate binary logistics regression analysis, age (p < 0.01), COPD (p < 0.05) and NLR (p < 0.05) remained independently associated with frailty (see Table 2).

**Table 2:** The relationship between CFS, clinicopathological characteristics, CT-BC and systemic inflammation, in patients with COVID-19 (n = 106)

	OR (univariate)	p-value	OR (multivariate)	p-value
Age	5.21 (2.10–12.9)	0.001	4.84 (1.71–13.1)	0.003
Sex	0.85 (0.37–1.99)	0.714	–	–
Hypertension	2.65 (1.49–9.52)	0.008	–	0.322
COPD	11.07 (1.42–86.5)	0.022	9.41 (1.00–88.2)	0.049
Type 2 DM	2.92 (1.00–8.48)	0.049	–	0.064
NLR	2.12 (1.27–3.55)	0.004	2.02 (0.08–3.78)	0.027

*COPD* chronic obstructive pulmonary disease, *DM* diabetes mellitus, *NLR* neutrophil: lymphocyte ratio

Odds ratio, 95% CI, p value

### Relationship between CFS, clinico-pathological characteristics, systemic inflammation and 30-day mortality

On univariate analysis, only NLR was significantly associated with 30-day mortality (OR 2.19, 95% CI 1.05–4.54, p = 0.036, see Table 3). Neither age (0.629), COPD (p = 0.70) or frailty (p = 0.298) were associated with 30-day mortality.

**Table 3:** The relationship between CFS, clinicopathological characteristics, systemic inflammation and 30-day mortality, in patients with COVID-19 (n = 106)

	OR (univariate)	p-value	OR (multivariate)	p-value
Age	1.30 (0.45–3.69)	0.629	–	–
Frailty (CFS > 3)	1.87 (0.57–6.11)	0.298	–	–
COPD	1.25 (0.40–3.89)	0.700	–	–
NLR	2.19 (1.05–4.54)	0.036	–	–

*COPD* chronic obstructive pulmonary disease, *NLR* neutrophil: lymphocyte ratio

Odds ratio, 95% CI, p value

## Discussion

While the association between frailty, determined by the Clinical Frailty Scale (CFS), and clinical outcomes in patients with COVID-19 is well recognised [4], the basis of this relationship remains unclear [19]. The results of the present study found that frailty was independently associated with age, COPD and systemic inflammation. However, it was not associated with other recognised prognostic factors such as nutritional status or body composition. Therefore, it may be that the prognostic value of frailty, in patients with COVID-19, is in part dependent on chronological age, pre-morbid lung function and a pre-existing systemic inflammatory response.

During the first wave of the pandemic, the Clinical Frailty Scale (CFS) was utilised in the U.K and other European countries to aid decisions on treatment escalation and ceiling of care for patients admitted with COVID-19 [3, 20]. However, frailty has been considered a dynamic, somewhat reversible, process with targeted intervention [21, 22]. As such, it is highly plausible that those admitted during the study period, who were acutely unwell with coronavirus may have present frailer than their pre-morbid baseline. Indeed, a loss skeletal muscle mass-one of many causes of functional impairment (a hallmark of frailty) [23]—has been associated with COVID-19 infection [24, 25]. However, to date there are no studies examining variations in frailty status before and after COVID-19 infection. As such, further study is required to determine if these changes are persistent, like the cognitive and functional impairments observed in patients admitted with severe sepsis [26].

Frailty is a spectrum that reflects the systemic, global burden of human aging and erosion of the patient’s homeostatic reserve [27]. Although the prevalence of frailty increases with advanced age, it is not exclusive to elderly patients [28, 29]. Indeed, Kastora and co-workers found that when adjusted for age, frailty as defined by CFS, was independently associated with increased mortality in patients with COVID-19. However, it is highly likely that those who are elderly would be frail, and vice versa. This is in keeping with the observations of the present study that found frailty remained independently associated with age on multivariate binary logistics regression analysis. Therefore, further studies with a range of age groups will be required to tease out the prognostic value of frailty in patients with COVID-19.

In the present study, frail patients (CFS > 3) admitted with COVID-19 were significantly more likely to be hypertensive, non-insulin dependent diabetics and have COPD compared to those who were not frail (See Table 1). This observation is consistent with recent work by Hanlon et al. who found that in a biobank of 493, 737 patients, that frailty was associated with co-morbidity [30]. Therefore, while the current literature suggests that frailty has prognostic value in patients with COVID-19 [4], it is of interest that diabetes mellitus [31], ischaemic heart disease [32] and COPD [33] have also shown an association with worsened outcomes. As such, it remains unclear whether frailty has independent prognostic value in patients with COVID-19 or is simply reflective of other prognostic domains such as co-morbid disease. Indeed, the impact co-morbidity has on the prognostic value of frailty in those with COVID-19 is exemplified in the work of Owen et al., who found that when adjusted for co-morbidity, frailty did not significantly impact mortality in elderly patients hospitalised with COVID-19 infection [8]. As such, further study is required to delineate if frailty remains prognostic in co-morbid patients with COVID-19 or is simply reflective of other prognostic domains.

While the basis of the relationship between frailty and clinical outcomes remains unclear, a recent review by Hussien et al. hypothesised that frail patients have a pre-existing immuno-pathological base, that puts them at a higher risk of mortality if they contract COVID-19 [19]. Indeed, a chronic inflammatory response and immunosenescence is recognised with advanced age [34, 35]. In the present study, systemic inflammation, as measured by NLR, was associated with frailty and 30-day mortality. The present observations are in keeping with recent cohort studies that found an elevated NLR was associated with negative outcomes in patients with COVID-19 [36, 37]. Therefore, it may be speculated that the prognostic value and treatment of the systemic inflammatory response will be greatest in older, frail patients with COVID-19. However, few studies to date have examined the relationship between frailty and systemic inflammation [35]. Therefore, it remains unclear if relationship between frailty and systemic inflammation in patients with COVID-19 is independent of age.

There are a number of limitations of this present study. Firstly, the small sample size and limited observations having the potential for sparse data bias. Secondly, CT imaging of the abdomen at the level of the third lumbar vertebra is not routine practice in patients admitted with COVID-19. These patients were identified from admission with a positive PCR test and their medical records retrospectively screened for CT-imaging facilitating body composition analysis within 3 months of their positive PCR date, in keeping with standard practice of our unit. As such this may introduce selection bias into the cohort. Nevertheless, the present study has the largest cohort to date exploring the relationship between frailty, malnutrition, CT-body composition measurements and systemic inflammation in patients with COVID-19 infection, providing a novel insight into the relationship of frailty and other recognised prognostic factors. Lastly, the Clinical Frailty Scale (CFS) is a subjective assessment and therefore subject to observer bias. However, high inter-rater agreement of frailty scoring in patients with critical illness, across a range of healthcare professionals, has been reported [38]. Therefore, it is plausible that the prevalence of frailty in the present study and the associations observed, are representative of patients admitted with COVID-19. Nevertheless, it would be of interest to compare frailty prevalence and associations across multiple screening measures, also shown to have prognostic value, in patients admitted with COVID-19 [39].

In summary, frailty was independently associated with age, co-morbidity, and systemic inflammation. However, the basis of the relationship between frailty and clinical outcomes in COVID-19 requires further study.

## Data Availability

Data will be made available following request to the senior authors.

## Abbreviations

BMI: Body mass index
CFS: Canadian Study of Health and Aging-Clinical Frailty Scale
COPD: Chronic obstructive pulmonary disease
COVID-19: Coronavirus disease
CRP: C-reactive protein
CT-BC: CT-body composition
CXR: Chest radiograph
DM: Diabetes mellitus
mGPS: Modified Glasgow Prognostic Score
NLR: Neutrophil: lymphocyte ratio
PCR: Polymerase chain reaction
SFI: Subcutaneous fat index (cm^2^/m^2^)
SMA: Skeltal muscle area (cm^2^)
SMD: Skeletal muscle radiodensity (HU)
SMI: Skeletal muscle index (cm^2^/m^2^)
TFA: Total fat area (cm^2^)
VFA: Visceral fat area (cm^2^)
WHO: World Health Organization
